# From the archives: Roles of microtubule-associated proteins in organelle movement, tip growth, and phragmoplast architecture

**DOI:** 10.1093/plcell/koae331

**Published:** 2024-12-18

**Authors:** Renuka Kolli

**Affiliations:** The Plant Cell, American Society of Plant Biologists; Sainsbury Laboratory, University of Cambridge, Cambridge, UK

Microtubules are the largest cytoskeletal filaments that regulate the shape and internal organization of the eukaryotic cell. They are composed of tubulin dimers polymerized around a hollow core and undergo rapid cycles of assembly and disassembly. They are intrinsically polar, with a fast-growing plus end and a slow-growing minus end. New microtubules are formed by a process called nucleation. In plants, nucleation occurs primarily on the walls of existing microtubules such that the nascent microtubules appear branched at particular angles. Many microtubule-associated proteins regulate microtubule dynamics by nucleation, stabilization, destabilization, capping, or cross-linking. Kinesins are ATP-dependent motor proteins that move along microtubules to transport cargoes such as vesicles and organelles. Their motility can be assessed in vitro by the gliding assays, wherein surface-immobilized motor proteins propel microtubules to glide over a glass slide. Investigations on microtubule-associated proteins provide insights into their roles during various cellular processes, as exemplified below.

## 2000: Pollen tube organelle movement

Once pollen lands on the stigma of a flower, it extrudes a pollen tube that rapidly elongates through the style to deliver sperm cells to the ovule for fertilization. Intense movement of organelles in the pollen tube ensures their proper distribution in the cytoplasm. While there is no doubt that the organelle movement is driven by the actomyosin system, the involvement of microtubules and their associated motor proteins has been debated over the past 3 decades (Cai 2022). Cai et al. (2000) reported evidence to support microtubule-based organelle movement. In search of microtubule-based motors present in tobacco pollen tubes, they isolated 3 polypeptides that co-sedimented with microtubules in the presence of AMP-PNP, a nonhydrolyzable ATP analog. The authors ascertained that one of them is a kinesin-related motor due to its specific detection with an antibody that targets the kinesin motor domain, microtubule-stimulated ATPase activity, microtubule binding properties similar to a conventional kinesin, and the ability to glide microtubules in vitro. Immunolocalization of this 90-kD kinesin (p90) indicated that it binds the organelles localized along microtubules in the cortical region of the pollen tube. Whether organelles do indeed move along microtubules in the pollen tube and the involvement of p90 in the process are yet to be determined.

## 2020: Protonema tip growth

During mitosis, microtubules completely reorganize and form the mitotic spindle to separate daughter chromosomes. This process in animal cells involves Kinesin-13 and Kinesin-8, which act as microtubule depolymerases and regulate microtubule length and chromosome movement. The Kinesin-13As in Arabidopsis and rice were also shown to depolymerize microtubules but have acquired different cellular functions: xylem secondary cell wall pattern formation and glume length regulation, respectively (Oda and Fukuda 2013; Deng et al. 2015). The other paralog, Kinesin-13B, present in these plants might function redundantly and/or be involved in mitosis (Fujikura et al. 2014). To investigate Kinesin-13 and Kinesin-8 roles during plant mitosis, Leong et al. (2020) knocked out their corresponding 3 paralogs in *Physcomitrium patens* and generated null mutants. In contrast to severe mitotic defects observed in the animal mutants, the moss *Kinesin-13* mutant displayed only mild mitotic defects, while the *Kinesin-8* mutant did not display any obvious mitotic defect. Furthermore, microtubule depolymerase activity was not detectable for either kinesin in vitro, while microtubule catastrophe-inducing activity by Kinesin-13 and microtubule gliding activity by Kinesin-8 were observed. To investigate reduced colony growth observed in the *Kinesin-13* mutant, the authors examined moss protonema filament growth. Moss protonema tip cells grow in a polarized manner, similar to the pollen tube of flowering plants. Within the tip cell's apical dome, microtubule plus ends converge into a dynamic focus that correlates with cell expansion (Hiwatashi et al. 2014). Interestingly, both knockout lines displayed positional instability of the microtubule foci in the tip cells of protonema filaments that preceded their wavy growth phenotype, while the control displayed stable microtubule foci and straight protonema tip growth (Fig.). Therefore, moss Kinesin-13 and Kinesin-8 are required for straight protonema tip growth. The functional equivalents of animal Kinesin-13 and Kinesin-8 are yet to be identified in plants.

**Figure.**
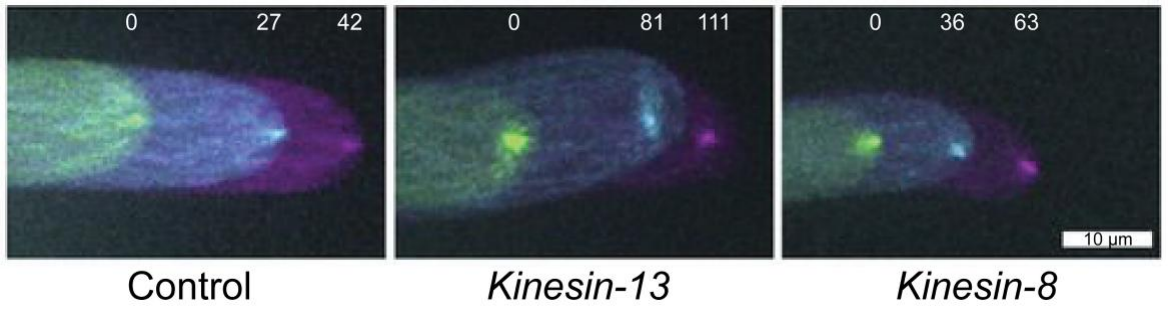
Fluctuating positions of microtubule foci result in wavy growth phenotype of protonema filaments in *Kinesin-13* and *Kinesin-8* mutants. Time-lapse images of GFP-tubulin fluorescence in maximum z-projections at specified time points in minutes are overlaid in different colors. Adapted from Leong et al. (2020), Figure 5C.

## 2024: Phragmoplast architecture

During late cytokinesis, a plant cell–specific cytoskeletal structure called the phragmoplast acts as a scaffold for cell plate assembly that will separate the 2 daughter cells. The phragmoplast forms in the center of the cell and expands centrifugally. Phragmoplast axial asymmetry is defined by faster microtubule dynamics in the midzone at the site of cell plate assembly than in the distal zone, where microtubules nucleate (Smertenko et al. 2011). Microtubule polarity with the plus ends directed toward the midzone is essential but not sufficient for axial asymmetry. Recently, Schmidt-Marcec et al. (2024) showed that microtubule nucleation plays a key role in phragmoplast axial asymmetry. Nucleation factors usually increase the polymerization rate or decrease the depolymerization rate and define the nucleation location. Based on transient overexpression assays, the 7 MACERATOR (MACET) paralogs from Arabidopsis function redundantly in decreasing the microtubule depolymerization rate. The authors attribute the loss of axial asymmetry observed in the double mutant lacking MACET4 and MACET5 to slower microtubule depolymerization in the midzone that was likely caused by a higher free tubulin pool that resulted from a lower rate of microtubule nucleation. The authors also found that MACET4 tethers AUG7, a subunit of the augmin complex, which initiates microtubule nucleation, to microtubules. Therefore, MACETs regulate phragmoplast axial asymmetry by directly promoting nucleation and indirectly reducing free tubulin availability. Furthermore, MACET4 and MACET5 appear to control the angle of branched microtubule nucleation at the leading edge of the expanding phragmoplast and regulate phragmoplast architecture.

## Dive Curated Terms

The following phenotypic, genotypic, and functional terms are of significance to the work described in this paper:

MACET4 Gramene: At1g23790
MACET4 Araport: At1g23790

MACET5 Gramene: AT1G70340
MACET5 Araport: AT1G70340

ATP CHEBI: CHEBI:15422

AUG7 Gramene: AT5G17620
AUG7 Araport: AT5G17620
